# Imaging in Primary Sjögren’s Syndrome

**DOI:** 10.3390/jcm9082492

**Published:** 2020-08-03

**Authors:** Martha S. van Ginkel, Andor W.J.M. Glaudemans, Bert van der Vegt, Esther Mossel, Frans G.M. Kroese, Hendrika Bootsma, Arjan Vissink

**Affiliations:** 1 Department of Rheumatology and Clinical Immunology, University of Groningen, University Medical Center Groningen, 9713 GZ Groningen, The Netherlands; e.mossel@umcg.nl (E.M.); f.g.m.kroese@umcg.nl (F.G.K.); h.bootsma@umcg.nl (H.B.); 2 Department of Nuclear Medicine and Molecular Imaging, University of Groningen, University Medical Center Groningen, 9713 GZ Groningen, The Netherlands; a.w.j.m.glaudemans@umcg.nl; 3 Department of Pathology and Medical Biology, University of Groningen, University Medical Center Groningen, 9713 GZ Groningen, The Netherlands; b.van.der.vegt@umcg.nl; 4 Department of Oral and Maxillofacial Surgery, University of Groningen, University Medical Center Groningen, 9713 GZ Groningen, The Netherlands

**Keywords:** primary Sjögren’s syndrome, imaging, salivary gland, sialography, salivary gland ultrasonography, magnetic resonance imaging, sialendoscopy, salivary gland scintigraphy, positron emission tomography

## Abstract

Primary Sjögren’s syndrome (pSS) is a systemic autoimmune disease characterized by dysfunction and lymphocytic infiltration of the salivary and lacrimal glands. Besides the characteristic sicca complaints, pSS patients can present a spectrum of signs and symptoms, which challenges the diagnostic process. Various imaging techniques can be used to assist in the diagnostic work-up and follow-up of pSS patients. Developments in imaging techniques provide new opportunities and perspectives. In this descriptive review, we discuss imaging techniques that are used in pSS with a focus on the salivary glands. The emphasis is on the contribution of these techniques to the diagnosis of pSS, their potential in assessing disease activity and disease progression in pSS, and their contribution to diagnosing and staging of pSS-associated lymphomas. Imaging findings of the salivary glands will be linked to histopathological changes in the salivary glands of pSS patients.

## 1. Introduction

Primary Sjögren’s syndrome (pSS) is a chronic, systemic, autoimmune disease characterized by dry mouth and dry eyes. As a heterogeneous systemic disease, many patients suffer from extraglandular symptoms, and almost all organs can be involved [1]. Because of the heterogeneity of the disease, pSS patients can present a broad spectrum of signs and symptoms, thereby making the diagnostic process challenging. The characteristic sicca symptoms of mouth and eyes, however, remain the most common manifestation of pSS. The dysfunction of the salivary glands and lacrimal glands is usually associated with chronic inflammation. For this reason, salivary gland biopsies are part of the standard diagnostic work-up, and the typical periductal lymphocytic infiltrates are an important criterion for pSS [2]. However, taking biopsies is an invasive surgical procedure and cannot be performed in all diagnostic centers. Imaging techniques, on the other hand, are noninvasive. It has been shown that imaging techniques can assist in the diagnostic process of pSS [3]. Imaging techniques could also be of value in assessing disease activity and detecting disease progression in pSS, which has already been shown in other systemic autoimmune diseases [4].

PSS patients have an increased risk of developing a non-Hodgkin’s lymphoma, mostly of the mucosa-associated lymphoid tissue (MALT) type. The prevalence of lymphoma development in pSS patients varies in different studies from 2.7% to 9.8% [5]. In pSS patients, MALT lymphomas most commonly arise within the parotid glands, but they can also develop at other extranodal locations, such as the lungs, lacrimal glands, or stomach [6,7,8]. Although the usefulness of imaging techniques in the diagnosis, staging, and treatment response evaluation in lymphomas in general is widely known [9,10], the value of imaging techniques in pSS-associated lymphomas is not yet clear.

In this descriptive review, we discuss the various imaging techniques used in pSS and link imaging findings to histopathological changes that occur in the salivary glands. We review the potential contribution of radiological and nuclear imaging techniques to the diagnostic work-up of pSS, and their role in assessing disease activity and disease progression. We also discuss imaging techniques that are currently used for the diagnosis and staging of pSS-associated lymphomas.

## 2. Diagnosis and Classification of pSS

Currently, there is no “gold standard” test or diagnostic criteria set to support diagnosis of this heterogenous and multisystemic autoimmune disease [11,12]. Therefore, diagnosis of pSS is still based on expert opinion, which relies on interpretation of a combination of several assessments. Although there is no consensus yet which assessments are necessary for diagnosing pSS, the diagnostic work-up can consist of different items, such as clinical examination, serological tests, oral and ocular tests, imaging techniques, and histopathology of the salivary gland. In the past years, multiple classification criteria sets were developed for pSS. These classification criteria were developed for research purposes, to allow selection of well-defined and homogenous populations of pSS patients for clinical studies. However, the terms diagnosis and classification in pSS are often used interchangeably since diagnosis and classification depend on similar items/tests. Table 1 shows the items that are included in the various classification criteria sets. In this review, we focus on the value of imaging techniques in the diagnostic work-up of pSS. When we specifically discuss imaging techniques as items of classification criteria sets, we add the term classification.

## 3. Histopathology of the Salivary Gland

**Figure 1:**
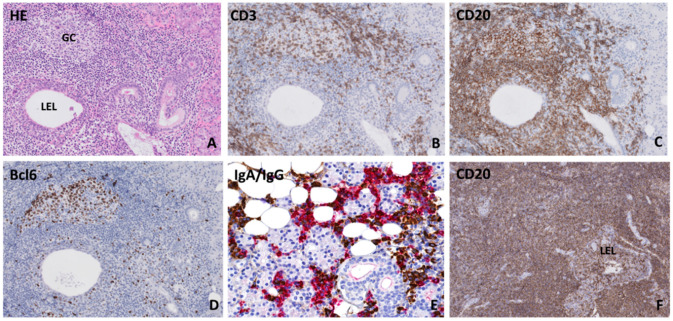
Histopathological features in parotid salivary glands of primary Sjögren’s syndrome patients (**A**) Lymphocytic infiltrate located around a hyperplastic striated duct (lymphoepithelial lesion: LEL) without obstructed lumen. Both (**B**) CD3+ T-lymphocytes and (**C**) CD20+ B-lymphocytes are present in the periductal infiltrate and within the ductal epithelium. (**D**) Presence of a germinal center, which was revealed by the presence of a cluster of ≥5 adjacent Bcl6-positive cells within a focus [28]. (**E**) Immunoglobulin A (IgA) (red) and immunoglobulin G (IgG) (brown) staining shows a plasma cell shift towards IgG plasma cells. (**F**) Salivary gland mucosa associated lymphoid tissue (MALT) lymphoma biopsy, which shows a diffuse CD20+ B-lymphocytic infiltrate around lymphoepithelial lesions in the absence of normal salivary gland parenchyma.

For decades, salivary gland histopathology has played a major role in diagnosing pSS. The characteristic finding within labial and parotid gland biopsies is the presence of infiltrates around striated ducts, mainly consisting of B- and T-lymphocytes. From the number of periductal foci (clusters of >50 lymphocytes) per 4 mm^2^, the focus score can be calculated, which is used in classification criteria sets for pSS [2,14]. Another scoring system is the grading system by Chisholm and Mason. In this grading system, stage 0, 1, and 2 indicate no, slight, or moderate infiltration with less than one focus per 4 mm^2^, respectively. Stage 3 and 4 correspond with a positive focus score (≥1 focus per 4 mm^2^) [17]. Besides the presence of periductal foci, other characteristic features can be found within the salivary glands of pSS patients, such as influx of IgG plasma cells and the presence of lymphoepithelial lesions (LELs) and germinal centers [18,19,20]. LELs are defined as hyperplastic ductal epithelial cells with infiltrating lymphocytes. LELs can eventually lead to complete obstruction of ducts (Figure 1). In addition to these characteristic features, proportions of fibrosis and acinar atrophy within salivary gland tissue are higher in pSS patients compared to controls [21,22]. There is no agreement yet whether fatty infiltration is age-associated or specific for pSS [23,24]. Besides their role in the diagnostic work-up of pSS, biopsies may also be used to assess prognosis (Table 2). Higher focus score is associated with higher European League Against Rheumatism (EULAR) Sjögren’s syndrome disease activity index (ESSDAI) scores, severe serological profiles, and an increased risk of lymphoma development [25,26]. PSS-associated salivary gland MALT lymphomas are diagnosed on histomorphological appearance (Figure 1) in combination with clonal analysis of immunoglobulin heavy chain (IGH) variable(V)-diversity(D)-joining(J) (VDJ) gene segments [27]. The following sections will discuss whether the characteristic histopathological findings correspond with imaging findings found in pSS patients.

## 4. Radiology Techniques

### 4.1. Sialography

**Figure 2:**
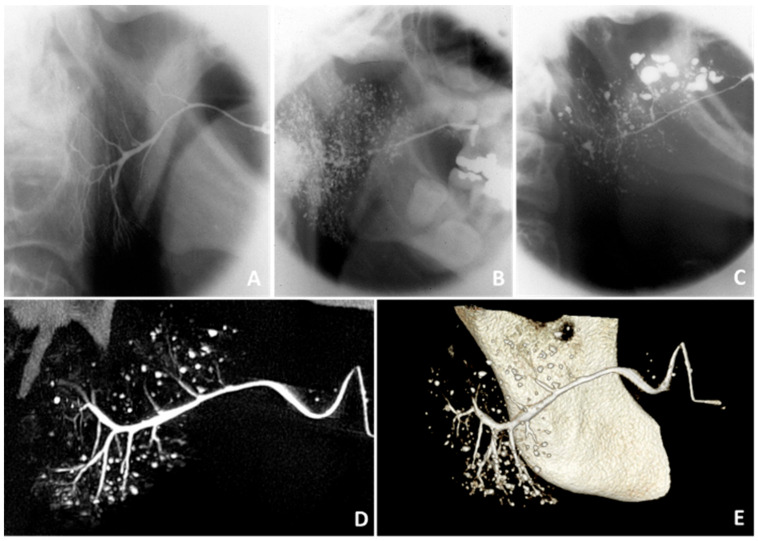
Findings on sialography. Sialographies of the parotid gland showing (**A**) no abnormalities in a healthy subject, (**B**) punctate/globular sialectasis in a pSS patient, and (**C**) globular/cavitary sialectasis in a pSS patient [29]. (**D**) Two-dimensional sialo-CBCT image and (**E**) three-dimensional sialo-CBCT image of the parotid gland of a pSS patient, showing normal width of the primary duct, moderate scarcity of ductal branches, and numerous diverse sialectasis. Thanks to Prof. D.J. Aframian and Dr. C. Nadler and colleagues who provided the sialo-CBCT images.

Sialography is a radiographic technique that visualizes the architecture of the ductal system by using X-ray projections after injection of contrast medium. In pSS patients, sialography shows sialectasis, which are collections of contrast material. The degree of sialectasis can be classified according to the scoring system developed by Rubin and Holt [29] (Figure 2). Sialectasis may be found at the location of cystic ductal dilatations in pSS patients. Another explanation could be that sialectasis represents extravasation of contrast material into the glandular parenchyma. A possible explanation for the leakage of contrast medium in pSS patients is dysfunction of tight junctions between striated ductal cells, due to the presence of proinflammatory cytokines [30]. In addition to sialectasis, sparsity of the ductal branching pattern can be found during sialography [3,31,32]. This could be due to obstruction of the ductal system, as a result of lymphocytic infiltration and proliferation of the ductal epithelium. However, direct associations with histopathological findings, such as the area of lymphocytic infiltrate or presence of LELs, have thus far not been reported.

Sialography has been used for diagnosing pSS for decades and shows moderate to high sensitivity and specificity [3]. This technique was excluded from the 2016 American College of Rheumatology/European League Against Rheumatism (ACR-EULAR) classification criteria (Table 1) because of multiple drawbacks. It is an invasive technique with risk of complications and radiation exposure. Furthermore, there are multiple contraindications like acute infection, acute inflammation, and contrast allergy [3,31].

Alternative sialographic techniques have been developed, such as sialo-cone-beam computerized tomography (sialo-CBCT) and magnetic resonance (MR) sialography. These techniques have an increased spatial resolution and provide three-dimensional, instead of two-dimensional, images of the ductal system. Keshet et al. [33] described correlations between sialo-CBCT findings and clinical data, such as xerostomia and serological parameters. However, since only 6 out of 67 sicca patients fulfilled the American European Consensus Group (AECG) classification criteria for pSS in this cohort, the usefulness of sialo-CBCT in pSS should be further investigated. MR sialography can identify changes within the salivary glands without the injection of contrast medium. The typical finding in pSS is the presence of multiple high-signal-intensity spots, which are thought to arise after leakage of saliva from peripheral ducts. Kojima et al. [34] did not find correlations between MR sialography findings and salivary flow rate, which can be explained by the fact that MR sialography visualizes the ductal system instead of saliva-producing acinar cells. Although MR sialography seems to be more sensitive to detect early disease, magnetic resonance imaging (MRI) provides more information on pathological changes in the glandular parenchyma, as we describe below [35].

In conclusion, sialography is not commonly used in the diagnostic work-up and follow-up of pSS anymore. Although alternative sialographic techniques such as sialo-CBCT and MR sialography have been evaluated, their current role in the diagnostic work-up of pSS is limited.

### 4.2. Magnetic Resonance Imaging

The role of MRI of the salivary glands in pSS has been investigated during the past decades. The characteristic finding in salivary glands of pSS patients is a heterogeneous signal-intensity distribution on T1- and T2-weighted images. The multiple hypointense and hyperintense areas cause a so-called salt and pepper appearance [34]. In the advanced stages of pSS, cystic changes can be found with MRI, which are thought to arise from destruction of the salivary gland parenchyma and the presence of fibrosis and fatty infiltration [3,31,36]. Although fat fractions in salivary glands seem to increase with higher age and body mass index [37] and can account for 60% of the histological section of the parotid gland in healthy individuals [38], imaging studies found that premature fat deposition found on MRI images is associated with SS [39,40]. Histopathological studies, however, did not make clear whether fatty infiltration is a specific feature of pSS or age-associated. Since biopsies do not represent the entire gland, it remains difficult to correlate MRI findings to histopathological findings. Although correlations between the focus score of the labial gland and MRI findings of the parotid gland were found [41,42], further associations between MRI findings and histopathological findings, such as the area of lymphocytic infiltration, fibrosis, and fatty infiltration, have not been investigated yet.

Although MRI showed added value in the diagnostic work-up of pSS by detecting pSS-specific abnormalities of the salivary glands, this technique is not routinely applied in pSS. Findings on MRI showed good agreement with salivary gland ultrasonography (SGUS). Since SGUS has several advantages over MRI, such as its high spatial resolution in superficial organs and the fact that SGUS is more easily accessible, SGUS is a better alternative for the diagnostic work-up of pSS [3,43].

Kojima et al. [34] demonstrated, in a group of pSS patients, that a higher degree of glandular heterogeneity and a smaller volume of the parotid and submandibular glands on MRI images were associated with lower stimulated and unstimulated salivary flow rates. These associations were even more pronounced for the submandibular glands, compared to the parotid glands, indicating that MRI findings of the submandibular glands can reflect hyposalivation. A possible explanation for the differences in associations between both glands is that the function of the submandibular gland is impaired earlier in the disease process than the function of the parotid gland [34,44,45]. Collection of saliva from the individual glands would be a more direct approach to relate MRI findings with salivary gland functioning in pSS. Similar to the MRI findings of Kojima et al. [34], lacrimal flow rates were associated with lacrimal gland volumes of pSS patients. Lacrimal flow rates were lower in pSS patients with atrophic lacrimal glands compared to patients with hypertrophic and normal-sized glands [46]. No studies have been performed to evaluate associations between MRI findings of the salivary glands and systemic disease activity, but MRI is the most appropriate imaging technique to evaluate central or peripheral nervous system involvement in pSS [47,48].

**Figure 3:**
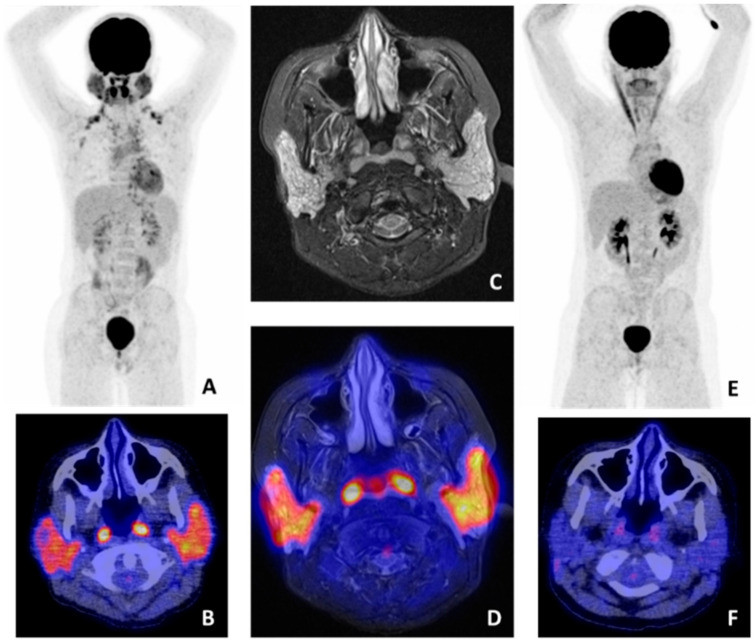
^18^F-fluorodeoxyglucose (FDG) positron emmison tomography/computed tomography (PET/CT) and magnetic resonance imaging (MRI) findings in a pSS patient with salivary gland mucosa associated lymphoid tissue (MALT) lymphoma. (**A**) Whole-body FDG-PET showing high heterogeneous FDG uptake in both parotid and submandibular glands. No other pathological lesions were found (axillary and clavicular regions with increased uptake represent brown fat). (**B**) FDG-PET/CT image showing pathological uptake in the parotid glands and physiological uptake in the tonsils. (**C**) MRI stir sequence showing a pathological, heterogeneous aspect of both parotid glands. (**D**) Manually fused FDG-PET/MRI image, showing pathological uptake in the parotid glands and physiological uptake in the tonsils. (**E**) Whole-body FDG-PET and (**F**) FDG-PET/CT image after treatment, showing no pathological uptake in the parotid glands, indicating complete remission.

MRI is also used for the evaluation of pSS-associated lymphomas in the head and neck region (Figure 3). MRI findings of salivary and lacrimal gland MALT lymphomas vary. Findings that have been described are glandular enlargement, (micro)cystic changes, and calcifications [49,50,51]. Zhu et al. [51] found that solid cystic appearances of MALT lymphomas can help to differentiate MALT from non-MALT lymphomas. However, benign and malignant lesions of salivary and lacrimal gland show overlap, which makes MRI a less reliable technique to differentiate between benign and malignant disorders of the exocrine glands [3,52,53]. Despite the indolent nature of pSS-associated lymphomas, these malignancies are able to disseminate to other mucosal sites or organs. MRI is used in local staging of the disease, by assessing the ingrowth in adjacent structures and spread to lymph nodes or other organs [50,54] (Table 2).

Together, MRI is not often used in the standard diagnostic work-up of pSS. However, due to its high spatial resolution, MRI is the most useful imaging technique for local staging of pSS-associated salivary and lacrimal gland lymphomas.

### 4.3. Salivary Gland Ultrasonography

Within the past decade, salivary gland ultrasonography (SGUS) has gained more and more attention, and was proven to be effective for the detection of typical structural abnormalities in pSS [55,56]. Furthermore, various studies demonstrated that addition of SGUS improves the performance and feasibility of the 2016 ACR-EULAR classification criteria [57,58,59,60]. However, many different SGUS-based scoring systems are available, and international consensus on which scoring system should be used is lacking. This hampers addition of SGUS to the classification criteria [55,56]. Therefore, the Outcome Measures in Rheumatology Clinical Trials (OMERACT) SGUS task force group has recently developed ultrasound definitions and a novel SGUS scoring system with good and excellent inter and intraobserver reliabilities, respectively [61]. Further studies should validate this scoring system before SGUS can be added to the 2016 ACR-EULAR classification criteria.

**Figure 4:**
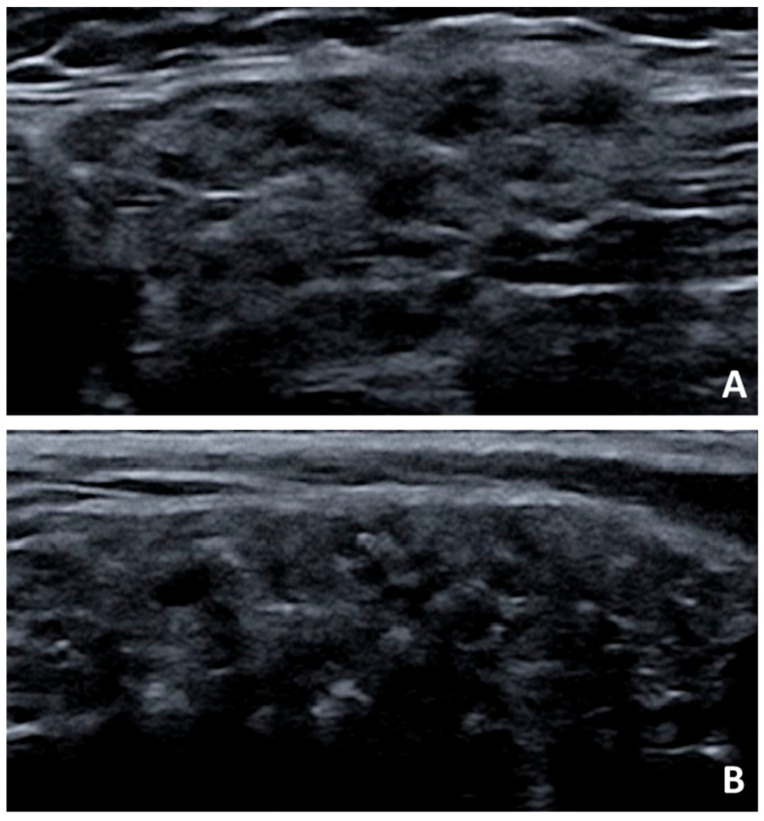
Salivary gland ultrasonography findings in pSS. Presence of hypoechogenic areas and hyperechogenic reflections in the (**A**) submandibular gland and (**B**) parotid gland of a pSS patient.

Typical ultrasonographic abnormalities in pSS are hypoechogenic areas, hyperechogenic reflections, and poorly defined salivary gland borders [55] (Figure 4). Mossel et al. [62] demonstrated that the presence of hypoechogenic areas is the most important SGUS feature. However, it is still unknown what these hypoechogenic areas reflect at a histological level. It has been suggested that hypoechogenic areas consist of foci containing inflammatory cells. Histopathological foci, however, are smaller in size compared to the hypoechogenic areas. Preliminary results of Mossel et al. [63] show a good correlation between hypoechogenic areas and percentages of CD45+ leukocytic infiltrate. These results indicate that, despite the differences in size, hypoechogenic areas are somehow associated with foci of inflammatory cells. One explanation for this association could be that hypoechogenic areas originate from leakage of saliva that is transported through the ductal system into the periductal infiltrate, and eventually into the salivary gland parenchyma. Leakage of saliva from the ductal system can be a comparable phenomenon to leakage of contrast medium during sialography, due to dysfunction of tight junctions between striated ductal cells [30]. However, collections of saliva in the periductal infiltrates or parenchyma are not commonly seen in salivary gland biopsies of pSS patients. Another hypothesis is that the hypoechogenic areas represent fatty infiltration. However, fat tissue is most often visible as a hyperechogenic instead of hypoechogenic area [64]. Furthermore, preliminary results of Mossel et al. [63] show poor associations between hypoechogenic areas and the percentage of fat cells in the total salivary gland parenchyma, which contradicts the latter hypothesis.

As described before, the area from which the parotid biopsies are taken may not be representative for the ultrasonographic images. The biopsy is taken from the periphery of the gland, which does not contain larger excretory ducts. Therefore, correlating histopathology to SGUS findings remains difficult. A possibility to get a better understanding of what SGUS features in salivary glands of pSS patients represent is taking ultrasound-guided core needle biopsies. A recent study by Baer et al. [65] showed that taking core needle biopsies in pSS patients suspected of salivary gland lymphoma is a safe and useful procedure. Although the morphology of core needle biopsies is inferior compared to that of open biopsies, the core needle method allows biopsies to be taken from the exact location of hypoechogenic areas or hyperechogenic reflections.

Another unresolved question is whether current SGUS scoring systems are sensitive enough to assess (treatment-induced) differences that are seen histopathologically. Current SGUS scoring systems use subjective categorical scales. Hypoechogenic areas, for instance, are scored on a 0–3 scale. Therefore, major changes need to occur in order to change from one category to another. This could be an important drawback when using SGUS as an objective tool to assess disease progression as well as to assess changes in salivary gland involvement in clinical trials [56,66]. Scoring SGUS findings on a continuous scale and in a more objective way could increase the sensitivity to change. A possible way to do this is by using image segmentation and artificial intelligence. HarmonicSS, a multicenter and EU-supported project, is applying artificial intelligence to SGUS images in pSS. Preliminary results show that, among the tested algorithms, the multilayer perceptron classifier is the best performing algorithm. Since the HarmonicSS cohort will increase in size over time, further validation will follow [56,67].

Despite the potential usefulness of SGUS in diagnosis and classification of pSS, the value of SGUS to assess disease activity and disease progression and to detect salivary gland MALT lymphoma needs to be established. SGUS scores seem to correlate with objective salivary gland function, as unstimulated salivary flow rates were found to be lower in SGUS-positive patients, compared to SGUS-negative patients [68,69,70]. Zabotti et al. [71] described that of all SGUS findings, the presence of hyperechogenic bands was independently associated with salivary flow rates. Although they suggested that damage of the glands is reflected by hyperechogenic bands, it is still unclear what these hyperechogenic bands reflect at a histological level. Several studies showed associations between SGUS scores and clinical parameters of disease activity, such as ESSDAI scores, IgG levels, and rheumatoid factor (RF) levels [68,72,73,74,75]. In contrast, other studies did not find correlations between SGUS scores and ESSDAI [69,76]. These discrepancies can be explained by differences in patient characteristics between cohorts, and by the fact that severe salivary gland involvement might not reflect systemic disease activity in all pSS patients.

Since previous studies found associations between SGUS findings and risk markers of lymphoma, such as cryoglobulinemia, lymphopenia, and persistent salivary gland swelling, Theander et al. [72] and Coiffier et al. [77] stated that SGUS can identify patients at risk of developing lymphoma. However, these results were found in retrospective cohorts, and longitudinal studies should be performed to assess whether SGUS findings are predictive of lymphoma [78]. Furthermore, the capability of SGUS to detect lymphoma compared to histopathology and MRI should be clarified.

The studies presented thus far provide evidence that SGUS has added value in the diagnostic work-up of pSS (Table 2). Further research should be performed on the development of a consensus scoring system. Furthermore, to assess the usefulness of SGUS in follow-up and lymphoma detection in pSS, longitudinal studies are needed. Several initiatives have started already, such as OMERACT and HarmonicSS projects, which will give us more insight into the potential of this imaging tool in pSS.

## 5. Sialendoscopy

Sialendoscopy is a minimally invasive technique used for both diagnosis and management of obstructive salivary gland disorders, such as sialolithiasis, anatomic ductal abnormalities and mucus plugs. With this gland-sparing technique, a sialendoscope is entered through the ductal orifice of major salivary glands for inspection and irrigation of the ductal system, after local or general anesthesia.

In pSS patients, sialendoscopic examination mainly shows strictures, but mucous plugs and a pale, minimally vascularized ductal wall of the larger excretory ducts can also be observed [79,80,81]. Ductal strictures can cause ductal obstruction and could therewith account for glandular swelling and pain in pSS. It is still unclear what these strictures reflect at a histological level. One hypothesis is that strictures are caused by large LELs that obstruct the ductal system, but no histological studies have been performed yet to prove this hypothesis. Since the sialendoscopic findings in pSS are not specific for the disease, the diagnostic value of this technique in pSS is limited. However, various studies show that dilatation of strictures in combination with irrigation of the ductal system with saline and/or corticosteroids by using sialendoscopy is a safe and effective treatment option for salivary gland dysfunction in pSS patients. Studies showed that both subjective and objective oral dryness improved after sialendoscopy [80,82,83]. Furthermore, visual analogue scale pain scores decreased after the procedure [79,84], and the number of episodes of glandular swelling declined after treatment [85]. However, the above mentioned (pilot) studies included relatively small numbers of patients. The clinical benefits for pSS patients after sialendoscopy should be further explored in larger cohorts.

Although complications, such as infections and postoperative pain, seem to be limited [86], multiple studies reported that the sialendoscopic procedure was not successful in all pSS patients because of technical issues [79,80]. The most common difficulties reported were problems with identification or dilatation of the papilla before introducing the sialendoscope, which occurred more often during sialendoscopy of the submandibular gland compared to the parotid gland. These problems might be associated with characteristic features of severe stages of pSS, such as the presence of extreme hyposalivation and atrophic changes. Using salivary gland ultrasonography to assess the stage of the disease was suggested to predict whether patients would benefit from sialendoscopic treatment [80]. It would be of value to study this suggestion.

In summary, although sialendoscopy has no added value in the diagnostic work-up of pSS, recent studies have drawn attention to the fact that rinsing the ductal system, a procedure that accompanies sialendoscopy, might be useful in the management of oral symptoms.

## 6. Nuclear Medicine Techniques

### 6.1. Conventional Nuclear Medicine

Nuclear medicine imaging uses specific radiopharmaceuticals to visualize (patho)physiological processes in the body. Imaging with a a gamma camera system that provides two-dimensional planar images forms the basis of conventional nuclear medicine. However, it is often difficult to determine the exact location of increased tracer uptake by using these two-dimensional images. Three-dimensional images can be created by collecting images from different angles around the patient. This technique, called single photon emission computed tomography (SPECT), leads to a higher contrast and improves sensitivity, compared to two-dimensional nuclear medicine. Combining SPECT with a low dose or contrast-enhanced CT scan enables determination of the exact location of the area with increased uptake.

#### 6.1.1. ^99m^Tc-Pertechnetate Scintigraphy

Scintigraphy of the major salivary glands is a nuclear imaging technique that evaluates salivary gland function by uptake and secretion patterns of the radioactive tracer Technetium-99m pertechnetate (^99m^Tc-pertechnetate). ^99m^Tc-pertechnetate is actively taken up by salivary gland epithelial cells, probably by using Na+/I− symporters, and secreted into the ductal lumen along with saliva [87]. The technique was included in previous classification criteria of pSS, in which a positive scintigraphy was defined as delayed uptake, reduced concentration, and/or delayed excretion of the tracer [16]. However, due to the low specificity and the inability to differentiate uptake failure from secretory failure, scintigraphy was omitted from the ACR-EULAR classification criteria [14,88]. Various studies stated that scintigraphic examination should focus on the degree of salivary gland dysfunction in pSS, instead of the differentiation between pSS and non-SS [31,89].

Several studies found a relationship between scintigraphic findings and severity of the disease. Brito-Zéron et al. [90] concluded that severe scintigraphic patterns were a prognostic factor for developing extraglandular manifestations. In a large retrospective study by Ramos-Casals et al. [89], patients presenting with severe involvement of the salivary glands according to the scintigraphic examination not only showed increased risk of developing serious extraglandular manifestations, but also a higher risk of developing lymphoma and a lower survival rate. The latter authors also reported that scintigraphic findings worsened during follow-up in 32% of patients. This subgroup of patients also had higher prevalence of high ANA titers, compared to patients with stabilization or improvement of scintigraphy [89]. Furthermore, scintigraphy was associated with histopathological findings within labial salivary glands of pSS patients, as scintigraphic parameters decreased significantly with higher stages of lymphocytic infiltrates graded by Chisholm and Mason’s grading system [91,92,93].

#### 6.1.2. Future Promising Scintigraphic Tracers

Another scintigraphic tracer studied in pSS patients is ^99m^Tc-EDDA/Tricine-HYNIC-Tyr(3)-Octreotide (^99m^Tc-HYNIC-TOC). This tracer binds to somatostatin receptors on cell membranes. These receptors were shown to be overexpressed in various inflammatory and autoimmune diseases, and were found on, among others, activated lymphocytes, endothelial cells, and the monocyte lineage in synovium of rheumatoid arthritis (RA) patients [94,95,96]. Anzola et al. [97] showed increased ^99m^Tc-HYNIC-TOC uptake within salivary glands of pSS patients compared to controls, as well as a considerably higher sensitivity of ^99m^Tc-HYNIC-TOC scintigraphy compared to conventional scintigraphy. Furthermore, they demonstrated that ^99m^Tc-HYNIC-TOC scintigraphy was able to identify joint involvement in a cohort of 62 pSS patients, of which many patients (87%) reported joint pain. Another tracer with potential applicability for pSS is ^99m^Tc labeled with rituximab, which is an anti-CD20 tracer that images B-lymphocytes. In a small experimental setting, this tracer showed variable uptake in salivary glands and moderate uptake in lacrimal glands in two pSS patients [98].

Together, ^99m^Tc-pertechnetate scintigraphy is in many institutes no longer used as an imaging technique in the diagnostic work-up and follow-up of pSS. Although promising new scintigraphic tracers have been developed, conventional nuclear medicine has disadvantages, such as the limited resolution of 8-10 mm and the inability to quantify the exact uptake. Positron emission tomography (PET), combined with low dose or contrast-enhanced CT, has multiple advantages over conventional scintigraphy, such as better spatial resolution, faster imaging tracts, and the possibility to quantify tracer uptake. It would be of value to couple the promising tracers that are mentioned above to a PET radionuclide in order to study the added value of these PET tracers in pSS patients.

### 6.2. Positron Emission Tomography/Computed Tomography

PET is an imaging tool developed in the 1990s to visualize specific (patho)physiological processes of a particular area or of the whole body. The technique is based on injection of radioactive tracers, which are tracers attached to radionuclides that emit positrons (positively charged electrons) to become stable. Emitting positrons cannot exist freely and annihilate with antimatter (negatively charged electrons) by emitting two gamma-ray photons, each with the same energy (511 keV) in direct opposite directions. The PET camera system consists of a ring-shaped detector system which can detect the two photons when they arrive within a certain time frame. Recent developments in software of PET cameras have led to a correction method for the time these photons need to travel to the detector, the so-called Time-of-Flight technique. This improvement (since 2005) caused a higher efficacy in detecting photons. Since then, the use of PET imaging for clinical and research purposes increased considerably. Although PET was originally used in oncological diseases to detect malignancies, the usefulness of PET to image infection and inflammation has markedly increased during the last decade, and PET techniques evolved rapidly. Hybrid camera systems were developed to combine PET findings with CT or MRI to add anatomical information. These new camera systems provide better spatial resolution images (around 3–4 mm), decrease scan duration and radiation dose, and lead to increased diagnostic accuracy. Furthermore, the development of guidelines to standardize PET/CT techniques between centers by the European Association of Nuclear Medicine (EANM) enhances comparability of data and promotes multicenter research [99,100]. In addition, new and specific tracers to image infectious and inflammatory diseases are constantly being developed.

#### 6.2.1. ^18^F-FDG-PET/CT

**Figure 5:**
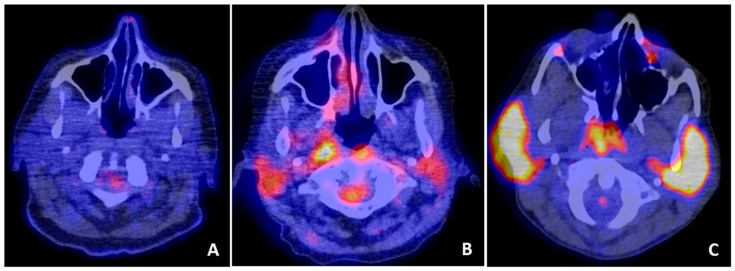
FDG-PET uptake patterns within the parotid glands. FDG-PET/CT showing (**A**) no increased uptake in the parotid glands of a subject without pathology in the head/neck region, (**B**) increased uptake in both parotid glands in a pSS patient, and (**C**) intense uptake in both parotid glands of a pSS patients with histologically confirmed parotid salivary gland MALT lymphoma.

The most commonly used PET tracer is ^18^F-fluorodeoxyglucose (FDG). Uptake of this tracer is relatively higher in cells that are metabolically active, such as inflammatory cells. FDG is indicated in several infectious and inflammatory diseases and is useful for the diagnosis and follow-up of various autoimmune diseases [4,101,102]. In pSS patients, several case reports showed abnormal FDG uptake in salivary glands [103,104,105] (Figure 5). However, physiological FDG accumulation in salivary glands is highly variable, and subjects without known head and neck pathology frequently show increased FDG uptake in the salivary glands [106,107]. A possible explanation for the wide range in physiological uptake is that salivary glands of different subjects utilize different amounts of glucose for metabolism [108,109]. Which human salivary gland cells have the highest glucose uptake is not known, but in rodent salivary glands, both acinar and ductal cells seem to play a role in glucose uptake [110,111].

Whether pSS patients show increased FDG uptake in inflamed salivary and lacrimal glands is not yet clear. Although Cohen et al. [112] reported increased FDG uptake in salivary glands of pSS patients compared to a control group of patients who underwent PET for an isolated pulmonary nodule, another study could not confirm this finding [36]. However, both studies used different scoring methods and camera settings, and standardized guidelines and EANM Research Ltd. (EARL) reconstructions were not applied in these studies.

Besides visualizing salivary gland inflammation, several authors reported the ability of FDG-PET/CT to detect systemic disease activity in pSS [104,105,112,113,114,115]. Abnormal FDG uptake was observed in 75–85% of pSS patients, mainly within salivary glands, lymph nodes, and lungs [112,113]. However, not all organ involvement in pSS can be visualized by FDG-PET/CT, such as neuropathies, cutaneous vasculitis, and other skin abnormalities, which is mainly due to limited spatial resolution of PET cameras. Since the optimal spatial resolution is around 3-4 mm in newer PET/CT systems, it would be of interest to use these newer systems to study the effectiveness of FDG-PET/CT in assessing systemic disease activity in representative pSS cohorts.

The applicability of FDG-PET/CT in MALT lymphomas is still controversial due to variable FDG avidity at different MALT locations. Pulmonary and head/neck MALT lymphomas seem to be most FDG avid, and FDG-PET/CT shows higher sensitivities at these regions [116,117]. Since pSS-associated MALT lymphomas frequently develop in the salivary glands and/or lungs, there may be a role for this technique in the detection of lymphomas associated with pSS [113,118,119] (Figure 3). Cohen et al. [112] reported a higher maximum standardized uptake value (SUVmax) in lymphoma patients compared to pSS patients without lymphoma. Since only four lymphoma patients were included in this retrospective cohort, the study was not powered to affirm the usefulness of FDG-PET/CT in the diagnosis of pSS-associated MALT lymphoma. In another retrospective study by Kerean et al. [113], 8 out of 15 pSS patients had confirmed salivary gland or pulmonary MALT lymphoma. They found that a SUVmax of ≥4.7 in the parotid glands and presence of focal lung lesions were associated with lymphoma. An important advantage of FDG-PET/CT is the possibility to detect extraglandular lymphoma locations by using whole-body imaging. In addition, Kerean et al. [113] showed that FDG-PET/CT is useful for biopsy guiding and for treatment response monitoring [113]. However, the mentioned SUVmax threshold found in this study cannot directly be used by others, as different nonstandardized camera systems were used in this multicenter study. Importantly, both retrospective studies state that the frequent presence of benign lymph node uptake in pSS patients can be misleading and should be taken into account when using FDG-PET/CT for the diagnosis of lymphoma in pSS patients [112,113].

#### 6.2.2. Future Promising PET Tracers

Similar to conventional scintigraphy, specific PET tracers can be used to visualize inflammation in pSS. A case report showed intense uptake of ^68^Ga-pentixafor, a radioligand of the chemokine receptor CXCR4. This receptor is involved in, among others, migration of leukocytes toward sites of inflammation. In this pSS patient, the increased uptake in salivary glands and lymph nodes was histologically proven to be attributed to inflammatory cell infiltration [120]. To further investigate the overexpression of somatostatin receptors in salivary glands and joints of pSS patients, as shown by ^99m^Tc-HYNIC-TOC scintigraphy, the specific PET tracer ^68^Ga-DOTATOC can be used [121]. Another pathological feature in pSS that could be visualized is the increased number of B-lymphocytes within salivary glands. Furthermore, by using whole-body B-lymphocyte imaging, not only B-lymphocytes within the salivary glands can be visualized, but also B-lymphocytes that are present in other organs associated with pSS. We previously showed that high numbers of B-lymphocytes within salivary glands of pSS patients predict response to rituximab (anti-CD20) therapy [122]. Whole-body imaging of B-lymphocytes could be an improved and noninvasive method to select pSS patients who are likely to respond to rituximab treatment. In rheumatoid arthritis and orbital inflammatory diseases, imaging of B-lymphocytes by using ^89^Zr-rituximab has shown potential in the detection of B-lymphocyte-mediated diseases, in the evaluation of rituximab treatment, and also in the selection of rituximab responders [123,124]. Therefore, it is of interest to study whole-body B-lymphocyte imaging by using PET/CT in pSS patients, and to assess whether this specific PET tracer can detect treatment response and can identify rituximab responders at baseline. Since T-lymphocytes are thought to be predominant in salivary glands in early stages of pSS [125], imaging of these lymphocytes could also be of added value. Radiolabeled interleukin-2 (IL-2) can detect activated T-lymphocytes within affected organs in pSS since IL-2 receptors are overexpressed on activated T-lymphocytes [126].

Overall, PET seems to be a promising imaging technique in pSS. Although the added value of PET in the diagnostic process of pSS remains to be shown, FDG-PET/CT was found to be useful in the assessment of disease activity, the systemic staging of pSS-associated lymphomas, and the evaluation of treatment response.

## 7. Conclusions

Currently, various imaging techniques are used in the diagnostic work-up and follow-up of pSS patients, but none of them are included in the current 2016 ACR-EULAR classification criteria. Although sialography and scintigraphy have been part of previous classification criteria sets, both techniques are not commonly used in the diagnostic work-up of pSS anymore. An imaging technique that has proven to be of added value in diagnosing and classifying pSS is SGUS. The next step is to incorporate a consensus SGUS scoring system into the 2016 ACR-EULAR criteria, after its validation in independent cohorts. In addition, longitudinal observational studies and clinical trials are needed to understand the usefulness of SGUS in assessing disease activity and disease progression in pSS. Several initiatives were started already, such as OMERACT and HarmonicSS, which will give us more insight into the potential of this promising imaging tool in pSS. Another emerging technique in the evaluation of salivary gland and systemic involvement in pSS is PET, combined with CT or MRI. Additional studies are needed to further elucidate the presumed role of FDG-PET/CT in pSS, by using larger and representative cohorts, standardized scanning procedures, and harmonization between centers. Besides, new pSS-specific PET tracers should be further explored since they may provide promising insights into pathological processes. Regarding imaging of pSS-associated lymphomas, findings on MRI, SGUS, and FDG-PET/CT should be compared to histopathological findings in order to investigate which imaging technique is most appropriate for the detection and staging of pSS-associated lymphomas. Furthermore, it is still not known what the various imaging findings in salivary glands of pSS patients represent at a histological level. Further research, for example, by performing ultrasound-guided biopsies, is needed to answer this question.

## Figures and Tables

**Table 1:** Comparison of classification criteria sets for pSS [13].

	2016-ACR-EULAR [14]	2012-ACR [15]	2002-AECG [16]
**ESSDAI ≥ 1**	+ (Entry Criterion)	−	−
**Sicca Symptoms**	+ (Entry Criterion)	−	+
**Salivary Gland Biopsy**	+	+	+
**Serology**			
Anti-Ro/SSA	+	+	+
Anti-La/SSB	−	+	+
Antinuclear Antibodies	−	+	−
Rheumatoid Factor	−	+	−
**Oral Signs**			
UWS ≤ 0.1mL/Min	+	−	+
Sialography	−	−	+
Scintigraphy	−	−	+
**Ocular Signs**			
Schirmer’s Test ≤ 5	+	−	+
Ocular Staining (OSS or vBv)	+	+	+

ACR-EULAR: American College of Rheumatology-European League Against Rheumatism; ACR: American College of Rheumatology; AECG: American European Consensus Group; ESSDAI: EULAR Sjögren’s Syndrome Disease Activity Index; UWS: unstimulated whole saliva; OSS: ocular staining score; vBv: van Bijsterveld score.

**Table 2:** Contribution of imaging techniques to the diagnostic work-up and follow-up of pSS patients.

	Contribution To:				Advantages	Disadvantages
	Diagnosing pSS	Assessing Disease Activity/Disease Progression	Diagnosing pSS -Associated Lymphoma	Staging pSS-Associated Lymphoma		
Salivary Gland Biopsy	+++	+	+++	-	-Gold Standard of Salivary and Lacrimal Gland MALT Lymphoma Diagnosis	-Invasive -Risk of Sampling Error
Sialography	+	+	−	−	-Moderate to High Sensitivity and Specificity	-Invasive -Contrast Medium
MRI	+	+	+	+	-High Spatial Resolution -Useful in Local Staging of PSS-Associated Lymphomas of Salivary and Lacrimal Glands	-Expensive -Moderate Differentiation Between Benign and Malignant Lesions of Salivary and Lacrimal Glands
Ultrasound	++	+	−	−	-Noninvasive -Widely Available	-No Consensus Scoring System
Sialendoscopy	−	−	−	−	-Possible Therapeutic Effect of Rinsing the Ductal System	-Invasive -No Added Value in Diagnostic Work-Up
Scintigraphy with ^99m^Tc-Pertechnetate	+	+	−	−	-Possibility of Whole-Body Imaging	-Low Specificity -Low Spatial Resolution
^18^F-FDG-PET/CT	+	++	+	+++	-Whole-Body Imaging -Useful in Assessing Treatment Response -Objective Quantification Possible	-Expensive -No Exact Interpretation Criteria for pSS Available

MRI: magnetic resonance imaging; PET/CT: positron emission tomography/computed tomography; MALT: mucosa-associated lymphoid tissue. Plus and minus signs are entered as follows: (+++) in case the imaging technique has an excellent contribution to the specific item, (++) for a good contribution, (+) in case the contribution is not yet clear or there is contradictive data, and (−) in case there is no evidence for contribution of the imaging technique to the specific item.
